# Prevalence of intestinal parasitic infection among children under 5 years of age at Dessie Referral Hospital: cross sectional study

**DOI:** 10.1186/s13104-018-3888-2

**Published:** 2018-10-29

**Authors:** Daniel Gebretsadik, Yeshi Metaferia, Abdurahaman Seid, Genet Molla Fenta, Alemu Gedefie

**Affiliations:** Department of Medical Laboratory Science, College of Medicine and Health Sciences, Wollo University, Dessie, Ethiopia

**Keywords:** Intestinal parasitic infection, Dessie, Under-five children

## Abstract

**Objective:**

Intestinal parasitic infection is a serious public health problem throughout the world particularly in developing countries. Like other countries in sub saran region epidemiological data regarding prevalence of intestinal parasites and their associated factors were limited in Ethiopia. So, the main objective of this study was to determine the prevalence of intestinal parasites and associated factors among under five children in Dessie Referral Hospital from August 1, 2017 to December 20, 2017.

**Results:**

In this research a total of 232 under five children were involved. Out of these study subjects 36 (15.5%) were infected with at least one intestinal parasites. A total of five intestinal parasites were examined and the dominant parasite was *E. histolytica* 15/232 (6.5%) followed by *H. nana* 11/232 (4.7%). All age groups were affected by intestinal parasites but children who were at the age of below 2 years and at the age between 2 and 3 years were 4.7 times and 2.6 times at risk of acquiring infection with intestinal parasites in comparison at the age of 3–5 years children.

**Electronic supplementary material:**

The online version of this article (10.1186/s13104-018-3888-2) contains supplementary material, which is available to authorized users.

## Introduction

Intestinal parasitic infection (IPI) is a serious public health problem throughout the world particularly in developing countries [1, 2], where the climate is suitable for spread the intestinal parasites [3]. According to WHO [4], soil-transmitted helminths (STH) are the second leading cause of mortality in children of age < 6 years who live in Africa. IPIs can cause symptoms like diarrhea, vomiting, loss of appetite, abdominal discomfort and an enlarged abdomen [5]. *Giardia duodenalis*, *Cryptosporidium parvum* and *Entamoeba histolytica* are the most common protozoan parasites that cause acute diarrheal illnesses among children [6]. Diarrhea is one of the consequences of IPIs among pre-schooled children and this may results in loss of fluid and electrolyte [7, 8].

In different parts of Ethiopia there were a number of research works that has been conducted on prevalence of IPI among children. A study done in southern part of the country revealed 26.6% prevalence. Six different types of IPs were detected and *E. histolytica* was the most commonly encountered parasite (11.4%) [7]. Six different types of IPs were examined in Wonji Shoa Sugar Estate. The overall prevalence was 24.3% and *H. nana* was the dominant IP (10.4%) followed by *S. mansoni* (8.8%) [9]. A study in North Shoa indicated 17.4% overall prevalence and the dominant parasite was *G. lamblia* (8.5%) followed by *E. histolytica* (5.7%) [10].

Many research works indicated different factors had association with prevalence of IPIs among under five children, some of these factors related with children practice and some others might be related with family particularly mothers’ practices. Children hand washing practice, mother’s educational status, nail trimming, drinking water from river source [10], age [7, 9], had associated with IPIs. Some other research works in Ethiopia revealed prevalence of IPIs did not show any association with gender [7, 9, 10].

Epidemiological information regarding the prevalence and associated factors of IPI and other diarrheal causing pathogens among under 5 years children is not available in many parts of the country including Dessie town (study area). Under five children need special care and follow up because they are more susceptible to intestinal parasites and other infectious pathogens due to their low level of immunity [11]. Therefore, the objective of this study was to assess the prevalence of IPIs and its associated factors among under-five children in Dessie Referral Hospital (DRH).

## Main text

### Methods

#### Study area, period and study subjects

A cross sectional study was conducted at DRH which found in Dessie, Ethiopia from August, 2017 to October, 2017. Dessie is located at latitude of 11°8′N and longitude of 39°38′E with an elevation between 2400 and 3200 meters above sea level and 401 km north east of Addis Ababa. The hospital catchment population is about 7 million and in 2016 the total non-bloody diarrhea cases were 2582. Out of these 1896 were children under 5 years of age. Under 5 years children who were attending the pediatric clinic of the hospital was the study subjects and those child who can provide stool sample and whose parent/guardian can give consent to be included in the study were eligible. Any children who were taking anti-helminthes within the last 2 weeks were excluded.

#### Sampling technique and sample size determination

Before starting the actual data collection we have assessed the average patient flow in each pediatric ward. We have randomly taken 20 children every day and included in the study. The sample size was determined using the single population formula. It was calculated by assuming a previous prevalence of 17.4% [10] with a margin of error of 0.05 and a confidence level of 95%. In line with it, 220 children were the minimum sample size.

### Ethical consideration

The study was conducted after obtaining ethical clearance from Wollo University research ethical committee. A written consent form was used to ask the willingness of the parent/guardian. Participants with positive IP result were communicated with the stake holders and treated in the pediatric ward of DRH.

#### Stool specimen collection and wet mount examination

Specimen was collected by clean, properly labeled and leak proof stool cup. After receiving the specimen the laboratory personnel examined by direct wet mount method using normal saline (0.85% NaCl solution) in the hospital Laboratory. The remaining sample was preserved with 10% formalin and examined by formol–ether concentration technique and modified Zeihl–Neelsen method at Wollo University teaching laboratory set up by investigators and experienced laboratory technical assistants.

#### Formol–ether concentration technique

For each stool specimen collected formol–ether concentration technique was performed. An estimated pea-size of faeces was emulsified in 4 ml of 10% formol water. Next another 4 ml of 10% v/v formol water was added and mixed well by shaking. Four ml of diethyl ether was added after sieving of the emulsified faeces. Then the tube was mixed for 1 min and immediately centrifuged at 750–1000*g* (3000 revolution per minute) for 1 min. After centrifuging, the parasites sedimented to the bottom of the tube and the faecal debris collected in a layer between the ether and formol water. Then, the sediment was transferred to a slide and covered with a cover glass. Finally the preparation was examined microscopically [12].

#### Modified Ziehl–Neelsen method

Smear from the remaining sediment was stained with Carbol fuchsin for 15 min and fixed with methanol for 2–3 min. The stain was decolorized with 1% acid alcohol for 15 s and counterstained with methylene blue for 30 s [12].

#### Data collection, processing and analysis

An interview based structured questionnaire was used to collect socio-demographic and other data from the parent or guardian of each study subject. The data (questionnaire based data) was collected by medical doctor professionals and instruction about the procedure of collection was provided by the investigators. Data quality was checked and entered to Microsoft Excel and exported to SPSS version 20 software and analysed. Binary logistic regression was done to investigate the relationship between the dependent and independent variables. P < 0.05 was considered statistically significant.

### Results

#### Socio-demographic characteristics

A total of 232 under five children had participated in this research. Of which 133 (57%) were male, almost half of the study participants were below the age of two, majority of them were urban dwellers and more than half of the guardians/parents were governmental employee (Table 1).

**Table 1:** Socio-demographic characteristics of children and their parents/guardians at DRH from August 2017 to October 2017

S. no.	Parameter	Number	Percent
1	Sex		
	Male	133	57.3
	Female	99	42.7
2	Age in year		
	< 2	110	47.4
	2–3	68	29.3
	3–5	54	23.3
3	Residence		
	Urban	165	71.1
	Rural	67	28.9
4	Parent or guardian occupation		
	Farmer	58	25
	Governmental	125	53.9
	House wife	12	5.2
	Merchant	34	14.7
	Others	3	1.3
5	Monthly income of guardian or parent (birr)		
	< 500	6	2.6
	501–1000	11	4.7
	1001–2000	45	19.4
	> 2000	170	73.3
6	Family size		
	≤ 3	76	32.8
	3–5	136	58.6
	> 5	20	8.6

#### Associated factors and parasitic infection

More than half the study participants were ceased breast feeding, majority of them initiated complimentary food after the age of 6 months, only few parent/guardian cannot even read and write (illiterate), almost half of the guardians/parents were washing their hands after utilization of toilet and before preparing food frequently (Additional file 1).

**Fig. 1:**
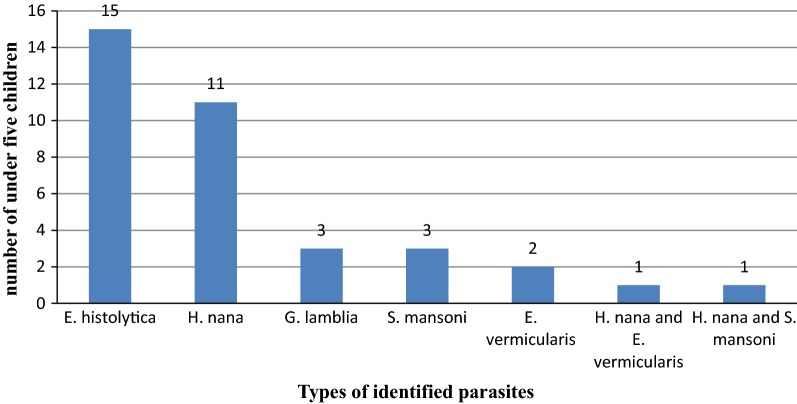
Identified intestinal parasites among under-five children, DRH, 2018

Of the total study participants, 36 (15.5%) were infected with one or more of IPs. The predominant identified parasite was *E. histolytica* 15/232 (6.5%) followed by *H. nana* 11/232 (4.7%). One mixed infection of *H. nana* and *E. vermicularis* and one mixed infection of *H. nana* and *S. mansoni* were identified (Fig. 1).

**Fig. 2:**
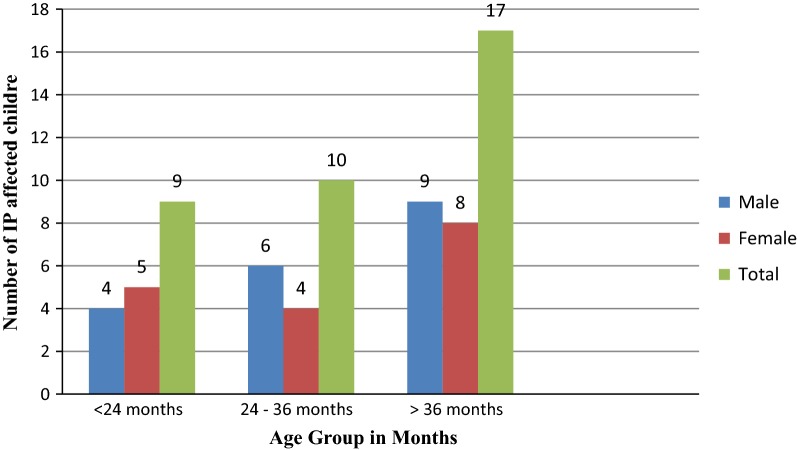
Age and sex distribution of children who were infected by IP, DRH, 2018

All age groups were affected by (IPs) but age groups of 3–5 years were highly affected one. There was slight difference between the number of IPs infected between male and female (Fig. 2).

In the binomial regression there were 6 variables that had association with prevalence of IPs. When we perform multinomial regression only one variable (age) was having association with the dependent variable. Children who were at the age of below 2 years and at the age between 2 and 3 years were 4.7 times and 2.6 times at risk of acquiring infection with IPs in comparison with 3–5.

*Entamoeba histolytica* which was the predominant parasitic infection affects all age of male study participants but it did not infect female children whose age group < 2 years of age. *H. nana* infection was slightly higher among female children than male and the parasite did not infect female study participants whose age group was between 2 and 3 years.

### Discussions

Assessing and investigating the distribution and extent of intestinal parasitic infection in a given community especially among vulnerable groups like children is a prerequisite for planning and evaluating intervention programs. The present study assessed the prevalence of intestinal parasitic (both Helminthes and protozoan) infections among under-fives children in the pediatric wards of Dessie Referral Hospital.

The overall prevalence of Intestinal parasite among 232 children under 5 years of age was 15.5% (36/232). *Entamoeba histolytica/dispar and H. nana* was the predominant parasitic infection and there was two study subjects who were affected by two parasites at the same time. Even though we were not assessing the hand washing habit of the child, in a given study children had poor hand washing practice and it makes them highly vulnerable to parasitic infections [10].

The prevalence of IPIs in our study was almost in agreement with a study that has been conducted in Debre-berhan (17.3%) [10], in Gondor [13] (17.3%), in Saudi Arabia [8] (17.7%) and Tanzania [14] (15.1%).

When we compare the current study with a study conducted in Wonji Shoa Sugar Estate, a higher overall prevalence (24%) of IPIs has been indicated [9]. This might be due to variation of place. The study participants may have the chance to contact with water bodies whereas most of our study participants had not such kind of exposure for water bodies. The other possible reason for the variation might be the method difference.

Another higher prevalence, 26.6% and 41.1%, of IPIs were reported in Hawassa [7] and Jimma [15], respectively. This might be due to difference in study subjects involved in the research works. In our case we were not including only children who were presenting with diarrhoeal diseases but also we were including children who did not have diarrhoeal disease complain. But in the above research works the study subjects were under five children who were presenting with diarrhoeal disease.

A much higher prevalence (85.1%) of IPIs was reported in a study that has been conducted in Southern Ethiopia [5]. Another higher prevalence (52.8%) of intestinal parasite infections was reported in the urban slums of Pakistan [16]. Most of our study subjects were urban dwellers and whose parents/guardian had relatively good financial status. This might be due to variation of study area and the method in comparison with our study.

Some studies indicated that the predominant parasite among children was *G. lamblia* [8, 10, 15, 16], where as in the current study the most encountered parasite was *E. histolytica*. *E. histolytica* was also the predominant parasite detected in a study conducted at Hawassa [7]. In the current study in two children double parasitic infection were detected where as a study conducted in Debre Birhan five [10] and in Hawassa six children were infected with two parasites [7].

Similar with our study there were some studies [7, 8, 16] that showed significance association of age with prevalence of IPIs. Like the current study, Studies in Ethiopia [9, 10] and Saudi-Arabia [8] indicated children of the age group 3–5 years were having the highest infection rate of IPs. On the other side it was in difference with a study conducted in Hawassa [7] that revealed the least infected age range was between 3 and 5 years.

Parents who have low level of education had risk of their children to acquire IPIs than other household heads who had higher education level with high statistically significant difference [8, 10]. But the current study did not show association between IPIs and parental education level.

### Conclusion

The overall prevalence of IPI among under five children was 15.5% and most study participants were affected by single IPs. The dominant parasite was *E. histolytica* followed by *H. nana* and only age variable had a significant association. Health information about how to prevent IPIs should be provided to parents. The hospital staffs especially the clinician should give special attention to diagnose the causes of children illness by using the utmost diagnostic facilities.

## Limitation

Due to budget shortage this study did not focus on bacterial and viral agents which are common infectious pathogens that can cause morbidity and mortality among children of under five.

## Additional file

**Additional file 1: Table S1.** Associated risk factors related information about the children and their parents/guardians at DRH from August 2017 to October 2017. **Table S2.** Binomial and multinomial regression results of independent variable with prevalence of IPIs among under five children at DRH from August 2017 to October 2017. **Table S3.** Sex, age and type of Parasite Cross-tabulation among under five children at DRH, from August 2017 to October 2017.

## Abbreviations

IPI: intestinal parasitic infection
DRH: Dessie Referral Hospital
