# Supplementary material for: Prevalence of intestinal parasitic infection among children under 5 years of age at Dessie Referral Hospital: cross sectional study
Source: BMC Res Notes. 2018 Oct 29;11:771. doi: 10.1186/s13104-018-3888-2 (PMC6206668; doi:10.1186/s13104-018-3888-2)
Supplement: Supplementary file 1 — Additional file 1: Table S1. Associated risk factors related information about the children and their parents/guardians at DRH from August 2017 to October 2017. Table S2. Binomial and multinomial regression results of independent variable with prevalence of IPIs among under five children at DRH from August 2017 to October 2017. Table S3. Sex, age and type of Parasite Cross-tabulation among under five children at DRH, from August 2017 to October 2017. [file 13104_2018_3888_MOESM1_ESM.docx]

**Table S1: associated risk factors related information about the children and their parents/guardians at DRH from August 2017 to October 2017**.

| s.no. | Parameter | Number | Percent |
| --- | --- | --- | --- |
| 1 | **Current breast feeding situation**  Exclusive breast feeding  Breastfed but also given complementary food  Ceased breastfeeding | 13  92  127 | 5.6  39.7  54.7 |
| 2 | **Complimentary food initiated**  Did not initiated  Before 6 months  After 6 months | 13  54  165 | 5.6  23.3  71.1 |
| 3 | **Parental or guardian education**  Illiterate  Read and write  Elementary  High school  College | 12  48  30  47  95 | 5.2  20.7  12.9  20.3  40.9 |
| 4 | **Parents/guardian hand washing practice**  Frequently  Infrequently | 114  118 | 49.1  50.9 |
| 5 | **Water source**  Pipe water in the compound  Pipe outside of compound  Unprotected source | 158  66  8 | 68.1  28.4  3.4 |
| 6 | **Animal in the house**  Yes  No | 63  169 | 27.2  72.8 |
| 7 | **Presence of dirty materials in the finger**  Yes  No | 39  193 | 16.8  83.7 |
| 8 | **Previous history of IP infection**  Yes  No | 62  170 | 26.7  73.3 |

**Table S2: Binomial and multinomial regression results of independent variable with prevalence of IPIs among under five children at DRH from August 2017 to October 2017**

| s.no. | Parameter | Number | P-value | COR (95% CI) | P-value | AOR (95% CI) |
| --- | --- | --- | --- | --- | --- | --- |
| 1 | **Current breast feeding situation**  Exclusive breast feeding  Breastfed but also given complementary food  Ceased breastfeeding | 13 (5.6)  92 (39.7)  127 (54.7) | 0.039  0.99  0.011 | 2.97 [1.3-6.8]  Ref. |  |  |
| 2 | **Age in year**  <2  2-3  3-5 | 110 (47.4)  68 (29.3)  54 (23.3) | 0.001  0.000  0.03 | 5.2 [2.1-12.6]  2.7 [1.1-6.4]  Ref. | 0.003  0.001  0.036 | 4.7 [1.9-11.5]  2.6 [1.06-6.41]  Ref. |
| 3 | **Eating of leftover food**  Yes  No | 51 (22)  181 (78) | 0.029 | 0.43 [0.2-0.92]  Ref. |  |  |
| 5 | **Animal in the house**  Yes  No | 63 (27.2)  169 (72.8) | 0.036 | 0.45 [0.22-0.95]  Ref. |  |  |
| 6 | **Dirty materials are there in the finger**  Yes  No | 39 (16.8)  193 (83.7) | 0.005 | 0.32 [0.14-0.71]  Ref. |  |  |
| 7 | **Parent or guardian occupation**  Farmer  Governmental  House wife  Merchant  Others | 58 (25)  125 (53.9)  12 (5.2)  34 (14.7)  3 (1.3) | 0.339  0.091  0.054  0.998  0.043  Ref | 15 [1.1-205] |  |  |

**Table S3: Sex, age and type of Parasite Cross-tabulation among under five children at DRH, from August 2017 to October 2017**

**Type of parasite Age (in month) Total**

**1-23.9 24-36 36.1-59.9**

***E. histolytica*** Male 3 3 3 9

Female 0 3 3 6

Total 3 6 6 15

***E.vermicularis*** Male 1 0 1

Female 1 0 1

Total 2 2

***G. lamblia*** Male 0 1 1

Female 1 1 2

Total 1 2 3

***H. nana*** Male 1 2 2 5

Female 3 0 3 6

Total 4 2 5 11

***H.nana* and**

***E.vermicularis*** Female 1 1

Total 1 1

***H.nana* and**

***S.mansoni*** Male 1 1

Total 1 1

***S. mansoni*** Male 2 2

Female 1 1

Total 3 3
